# A Methodology for Multivariate Investigation on the Effect of Acrylate Molecular Structure on the Mechanical Properties and Delivery Efficiency of Microcapsules via In Situ Polymerization

**DOI:** 10.3390/polym15204158

**Published:** 2023-10-19

**Authors:** Mattia Collu, Edoardo Rossi, Marta Giamberini, Marco Sebastiani, Rita Del Pezzo, Johan Smets, Edoardo Bemporad

**Affiliations:** 1 The Procter & Gamble Company, Temselaan 100, 1853 Strombeek-Bever, Belgium; delpezzo.r@pg.com (R.D.P.); smets.j@pg.com (J.S.); 2 Department of Civil, Computer Science and Aeronautical Technologies Engineering, Roma Tre University, Via Vito Volterra 62, 00146 Rome, Italy; edoardo.rossi@uniroma3.it (E.R.); marco.sebastiani@uniroma3.it (M.S.); edoardo.bemporad@uniroma3.it (E.B.); 3 Department of Chemical Engineering, Universitat Rovira i Virgili, Avda Paisos Catalans 26, 43007 Tarragona, Spain

**Keywords:** microencapsulation, nanoindentation, delivery efficiency, structure–performance correlations

## Abstract

In the field of encapsulation, microcapsules containing perfume have emerged as effective vehicles for delivering active ingredients across various applications. The present study employed a multivariate analysis framework to examine polyacrylate microcapsules for household products synthesized using different acrylate monomers. The advanced multivariate approach allowed us to quantify critical properties such as the *Molecular Weight between Cross-links* (*MW_c_*), mechanical attributes, *Encapsulation Efficiency* (*EE*), and *On-Fabric delivery*. It is worth noting that the mechanical properties were gauged using a novel nanoindentation technique, which measures the *Rupture Force per unit diameter* (*RFD*). Both *Encapsulation Efficiency* and *On-Fabric delivery* were assessed using GC-MS. Our findings identified the optimal microcapsule system as one synthesized with 100% aromatic hexafunctional urethane acrylate, showcasing a 94.3% *Encapsulation Efficiency* and an optimal *RFD* of 85 N/mm. This system achieved an exemplary *On-Fabric delivery* rate of 307.5 nmol/L. In summary, this research provides crucial insights for customizing microcapsule design to achieve peak delivery efficiency. Furthermore, by designing acrylic monomers appropriately, there is potential to reduce the amount of active ingredients used, owing to enhanced delivery efficiency and the optimization of other microcapsule properties. Such advancements pave the way for more environmentally friendly and sustainable production processes in the fast-moving consumer goods industry.

## 1. Introduction

Fragrances, essential in consumer goods, present challenges such as volatility and chemical instability [1,2,3]. Microencapsulation emerges as a solution, enhancing stability and controlled release in diverse applications, from personal care products to textiles [4].

The wall composition of these microcapsules, which can encompass materials such as polyamide and polyurea, is pivotal [5]. In this context, **our study leverages acrylate monomers, recognized for their beneficial properties** [6,7].

**The mechanical properties of perfume microcapsules are paramount**. They dictate the precise content release at the desired target, ensuring no premature rupture [8]. The wall material’s molecular structure profoundly influences these properties. Polymer concentration, solubility, and solvent removal rate are crucial in encapsulation efficiency [9].

Several studies have focused on the mechanical properties of microcapsules. For instance, Vinogradova and Radtchenko explored the role of wall materials and their microstructures on system performance [10,11]. Relevant to our scope, Yeo et al. emphasized the importance of optimizing rupture load in drug delivery microcapsules, demonstrating that a low polymer concentration could result in poor mechanical properties and inefficient drug release [12].

Nonetheless, although extensive research on microcapsules exists, the connection between the chemical structure of the capsule wall and the mechanical properties and delivery efficiency of microcapsules has received comparatively less attention. Indeed, a comprehensive understanding of the **relationships between the molecular structure of the capsules and their performance remains a crucial research gap**.

Our study introduces an innovative multivariate approach to address this research gap and the limitations of traditional methods. **Our primary objective is to establish correlations between the molecular structure and the mechanical properties, specifically focusing on the link between mechanical properties and delivery efficiency** in products like Liquid Fabric Enhancers.

Hence, we employed various acrylate monomers to craft capsule walls with distinct chemical structures and analyzed their delivery efficiency, among which the characterization of mechanical properties is a distinctive feature within this multivariate approach.

State-of-the-art experimental techniques for analyzing microcapsules’ mechanical properties include optical tweezers, shear flow apparatus, micropipette aspiration, micromanipulation, and atomic force microscopy [13,14]. Often combined with mathematical solutions like finite element modeling, these methodologies shed light on the stress–strain relationships in core–shell structures. However, while FEM has advanced our understanding, it often falls short in simulating rupture dynamics. Given the importance of rupture in encapsulation and delivery, our research emphasizes a novel methodology grounded in experimental validation. This approach addresses the complexities often overlooked by FEM and traditional methods, which often lack the sensitivity and precision required for nuanced analysis.

Some authors, moreover, have investigated the coupled effects of encapsulation and mechanical properties for specific polymeric core–shell systems. Valuable examples include, for instance, the work from Fernandes et al., who highlighted how the release of encapsulated perfume dye was demonstrated to be a function of deformation [15], and Su et al., who investigated Young’s modulus and the hardness of microcapsules within loading ranges that did not exceed the ‘yield point’ or ‘rupture point’ [16].

In this study, we advocate for the innovative use of nanoindentation for precise characterization [17]. This technique offers a high-resolution approach, measuring the force required for capsule rupture. It stands out as a method that can provide insights into the mechanical behavior of microcapsules under compression, a facet scarcely explored in the existing literature [18]. Thus, a novel nanoindentation-based methodology has been developed for high-throughput automated testing of fragrance-filled microcapsules. The technique is designed to produce vast amounts of statistically consistent data for a comprehensive multivariate analysis, allowing it to discern intricate relationships between the capsules’ properties, microstructures, and performances.

Through this investigation, we anticipate a deeper understanding of how the polymeric wall’s molecular structure relates to the microcapsules’ mechanical properties and On-Fabric delivery. The findings of this study will provide valuable insights into the design of microcapsules by leveraging molecular design to tune their delivery efficiency.

## 2. Materials and Methods

### 2.1. Raw Materials

In the context of the synthesis of microcapsules, the commercially available monomers aromatic hexafunctional urethane acrylate, dipentaerythritol penta-/hexa-acrylate, and 1,2-(oligoglycolate diacrylate) ethylene glycol (herein referred to as monomer A, monomer B, and monomer C, respectively) were used as-purchased without any further purification. Sartomer Americas (Exton, PA, USA) provided monomer A; Sigma-Aldrich (St. Louis, MO, USA) provided monomer B, while monomer C was synthesized by Ecosynth (Deinze, Belgium). 2,2′-Azobis(2-methylbutyronitrile), 2,2′-Azobis(2-methylpropionitrile), 4,4′-Azobis(4-cyanovaleric acid), isopropyl myristate (IPM), and polyvinyl alcohol (PVA) (85,000–124,000 g/mol) were provided by Sigma Aldrich (St. Louis, MO, USA).

Fragrance mixture A, consisting of a mixture of perfume raw materials with a volume-weighted average logP of 3.4 and *LFE* (Liquid Fabric Enhancer), was provided by The Procter & Gamble Company (Strombeek Bever, Belgium). 

IEC A Base detergent was supplied from WFK Testgewebe GmbH (Brüggen, Germany) to measure the *On-Fabric delivery*.

### 2.2. Synthesis of Microcapsules 

The acrylate monomers employed are listed in Table 1, where their MW is calculated as the average number of acrylate moieties per molecule. Monomer A consists of an aromatic hexafunctional urethane acrylate, monomer B is Dipentaerythritol pentaacrylate, while monomer C is 1,2-(triglycolate diacrylate) ethylene glycol.

The detailed composition of the batches under investigation is shown in Table 2. The monomer mixture employed is the sole parameter that differed in making the various samples. Precisely, the monomer composition and weight proportion variations are introduced to establish a structure–performance correlation.

For the sake of clarity, the names of the samples are defined as follows: CAP|A|B|C|, where A, B, and C correspond to the weight ratio of Monomer A, Monomer B, and Monomer C.

The core/wall ratio for all the samples equals 95/5, where the core is defined as the weighted sum of the fragrance and IPM, while the wall is defined as the sum of acrylic monomers.

**Figure 1:**
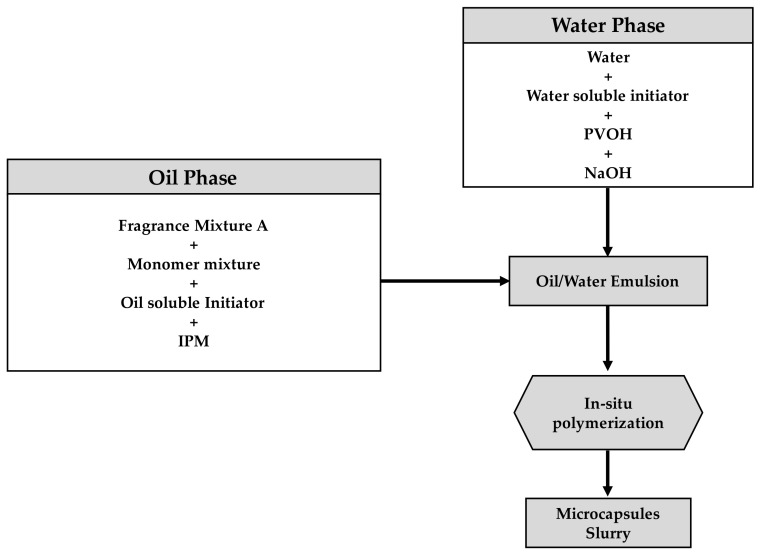
Workflow for the synthesis of microcapsules via in situ polymerization.

The capsules were synthesized via an in-situ polymerization process [19]. It consists of the dispersion of one phase containing a reactive monomer into a second immiscible phase [20]. Hence, two immiscible phases were prepared: water and oil phases, as shown in Figure 1.

A preliminary oil phase, consisting of 42.9 g of fragrance mixture A and 5.84 g of the acrylate monomer mixture reported in Table 2, was mixed until the monomer was fully dissolved. A second oil phase consisting of 26.9 of fragrance mixture A, 50.21 g isopropyl myristate (IPM), and 1.8 g of oil-soluble initiators (precisely 1.0 g 2,2′-azobis[2-methylbutyronitrile] and 0.8 g 4,4′-2,2′-Azobis[2-methylpropionitrile]) was prepared separately and mixed until complete homogenization. The two oil phases were then mixed at 500 rpm with an overhead stirrer Eurostar 200 digital from IKA (Staufen, Germany) using a 90-degree 4-blade stirrer [19].

The water phase, containing 178.6 g of a 2% water PVOH solution, 0.67 g of the water-soluble initiator (4,4′-azobis[4-cyanovaleric acid]), and 0.79 g of 21.5% NaOH, was prepared and mixed with an IKA Eurostar power control visc E20-MX-1 at 3000 rpm until the 4,4′-azobis[4-cyanovaleric acid] dissolved.

The water phase was added to the oil phase, and high shear agitation was applied to produce an oil-in-water (O/W) emulsion using an overhead stirrer Eurostar 200 digital from IKA (1200 rpm) with a 90-degree four-blade stirrer for 30 min. The emulsion was included in a glass jacketed flask reactor vessel of 500 mL equipped with a reflux condenser and overhead anchor stirrer. The temperature was increased to 75 °C in 30 min, held at 75 °C for 4 h, increased to 95 °C in 30 min, and held at 95 °C for 6 h. Finally, the batch was allowed to cool to room temperature. As a result, a population of microcapsules dispersed into the aqueous phase was obtained, which is herein defined as capsule slurry.

**Figure 2:**
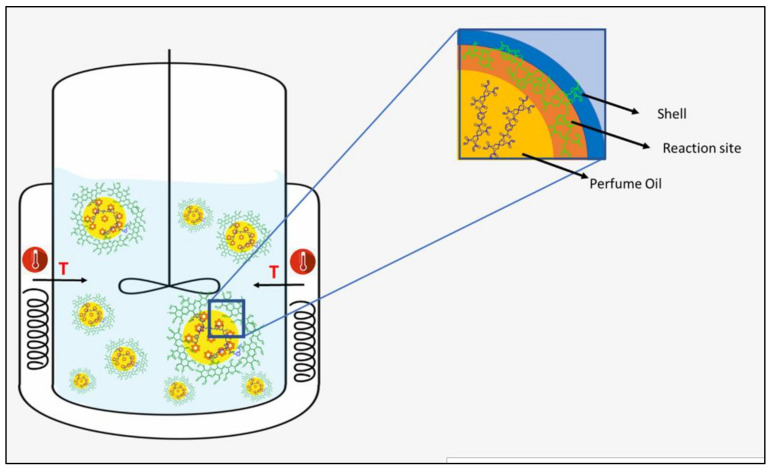
Schematic representation of wall formation via in situ polymerization.

As represented in Figure 2, the wall’s growth occurs within the oil phase, as the monomer is a component of the oil phase. The in-situ polymerization approach provides a unique and potentially more effective mean of achieving desired encapsulation outcomes than traditional interfacial polymerization commonly documented in the scientific literature, where the monomer is typically present in the water phase [21]. Within this specific process, the mass transfer of the acrylate monomers to the water/oil interphase is essential for successful encapsulation. Hence, the role of IPM is vital to optimize the partition coefficient of the core materials and, as a result, improve the mass transfer of monomer to the interphase.

### 2.3. HSP Distance to Characterize Monomer Solubility

During capsule synthesis, it is essential to characterize the solubility of the monomers in the fragrance mixture since the solubility impacts the transfer of monomers, which, in turn, impacts the quality of the capsule’s properties. The solubility of monomers is determined using the Hansen solubility parameters (*HSP*), which consider the combined effects of dispersion forces ($\delta_{d}$), polar forces ($\delta_{p}$), and hydrogen bonding forces $\;(\delta_{h}$) [22]. For each of the monomer mixtures employed, the distance in Hansen solubility parameters between the encapsulated fragrance mixture and the monomer mixture itself ($HSP\;Distance_{i}$) is defined via Equation (1):

(1) $$HSP\;Distance=\sqrt{4{\left(\delta_{d_{M}}-\delta_{d0}\right)}^{2}+{\left(\delta_{p_{M}}-\delta_{p0}\right)}^{2}+{\left(\delta_{h_{M}}-\delta_{h0}\right)}^{2}}$$

where $\delta_{dM}$, $\delta_{pM}$, and $\delta_{hM}$ are the mass-weighted *HSP* parameters for the monomer mixture, while $\delta_{d0}$, $\delta_{p0}$, and $\delta_{h0}$ are the mass-weighted *HSP* parameters for the fragrance mixture. The lower the distance in the Hansen space between the two mixtures, the higher the degree of miscibility.

The HSP parameters were calculated via HSPiP 5.4.08 software.

To preserve the fragrance composition’s confidentiality, each sample’s *HSP Distance* was normalized to the *HSP Distance* of a reference sample (CAP|1|0|0|), which was defined in Table 2.

### 2.4. Micro-Mechanical Compression Testing

Historically, techniques for testing the mechanical properties of microcapsules, as reviewed by Gray et al., while comprehensive, often relied on intricate compression geometries to discern the microcapsules’ behaviors, from elasticity upon contact to failure behaviors. Moreover, finite element modeling (FEM) has been instrumental in extrapolating stress–strain relationships for core–shell structures [13,14]. Notable works, such as Mercadé-Prieto et al., have provided insights into microcapsules’ mechanical characterization and failure stress estimation [23]. However, while these methods have advanced our understanding, they often lack the precision and sensitivity required for nuanced analysis.

Transitioning to our approach, we recognized the need for a more refined and precise methodology. Nanoindentation measurements were performed using a nanoindenter iNano (KLA Corp., Milpitas, CA, USA) equipped with an inForce50 actuator (KLA Corp., Milpitas, CA, USA) to measure the compressive strength of the hollow polymeric microspheres. The experimental setup utilizes the nanoindenter actuator with a flat punch diamond tip with a diameter of 100 μm, which applies controlled compression to the microspheres until rupture occurs.

The microcapsule slurry was diluted in 5 mL of deionized water with a dilution ratio of 1:1000. Small droplets of the diluted solution were deposited on laboratory slides previously glued on top of nanoindentation stubs with hot mounting compounds. The solvent evaporated before testing.

The compression process consists of two distinct phases. In the initial phase, the displacement of the punch is controlled, ensuring precise measurement and control of the applied force.

The surface identification procedure begins with the initial determination of the substrate position in an area adjacent to the microsphere of interest. Once the substrate position is identified, the system is positioned above the microsphere, and the indenter tip oscillates at the resonant frequency specific to the nanoindentation system being used. These oscillations are performed under force control, with an amplitude of approximately ±10 nm around zero position.

The compression phase is conducted without imposed oscillation, and rupture is identified through the derivative of the load signal. Rupture manifests as a sudden change in the load–depth curve. In load-controlled instruments, this sudden variation corresponds to a rapid change in displacement, while in displacement-controlled instruments, it corresponds to an immediate change in the applied load. The employed instrument implements a pseudo-displacement-controlled protocol between the two characteristics above.

**Figure 3:**
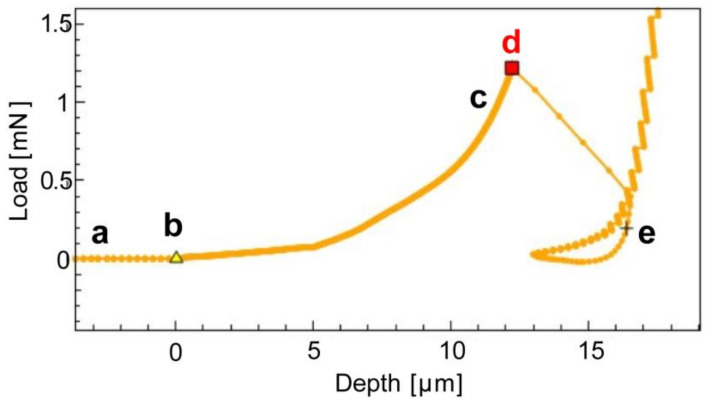
Load vs. Depth compression test of a single microcapsule. Stages comprise: (a) microcapsule approach by nanoindenter tip; (b, c), the tip comes into contact with the microcapsule, leading to a subsequent increase in load as the microcapsule is compressed; (d) the microcapsule ruptures; Subsequently, at point (e), the tip compresses the substrate, resulting in an exponential rise in Load.

**Figure 4:**
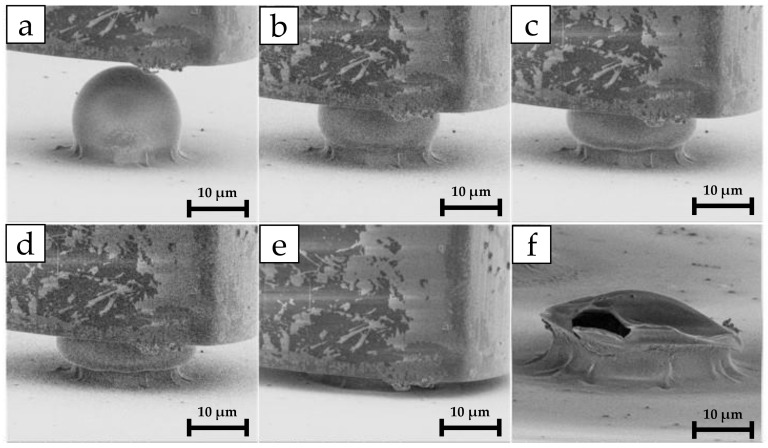
In situ nanoindentation time-lapse of the capsule compression: (**a**) flat-punch indenter approaching microcapsule; (**b**,**c**) compression stages; (**d**) rupture event and capsule structural integrity collapse; (**e**) substrate loading; (**f**) microcapsule post-mortem image with fracture view. The rupture force corresponds to the maximum load applied just before rupture, which in the load vs. depth graph of Figure 3 corresponds to point (**c**).

Figure 3 illustrates the relationship between load and depth as the compression of a single microcapsule progresses. The different point labels are linked to the time-lapse of the compression, which is presented in Figure 4.

To determine the diameter of the microsphere, a mechanical approach is utilized. The displacement position at which the surface is identified is compared to the displacement position at which the substrate is initially detected.

Compression tests are conducted at a constant velocity of 2 µm/s. This uniform velocity ensures that any potential effects related to strain rate on the behavior of the polymeric membranes, which are sensitive to such factors, are decoupled and minimized. The compressive strength of the microspheres is ultimately determined by various factors, including the force exerted by the contents encapsulated within the microsphere, the chemistry and microstructure of the polymer membrane, and the thickness of the membrane.

### 2.5. Formulation into Household Products

The microcapsules were formulated in a Liquid Fabric Enhancer (LFE) to evaluate the delivery efficiency. The microcapsules were added to the LFE to achieve a final perfume concentration of 0.3% through the use of microcapsules. The concentration of perfume in the microcapsules was defined as the percentage in weight of active (perfume) to the total weight of the microcapsule slurry. The composition of the LFE is shown in Table 3.

The Softening active is a diester quaternary ammonium compound (Ci-DEEDMAC = Ditallowoyl Ethoxy Ester Dimethyl Ammonium Chloride [MDEA based, Methyl Di-Ethanol amine based quat, available from Evonik, (Essen, Germany)]).

### 2.6. Encapsulation Efficiency Determination

The percentage of *Encapsulation Efficiency* in the microcapsule slurry was determined via gas chromatographic mass spectrometric analysis. Solid-phase microextraction (SPME) (50/30 µm DVB/Carboxen/PDMS) was employed.

Following the procedure outlined in the Formulation into Household Products section, two LFE products were made: *microcapsules in LFE* and *reference oil in LFE*.

The *microcapsules in the LFE product* were made by adding the capsule slurry to LFE, resulting in a perfume weight fraction of 0.3%. The *reference oil in LFE* was prepared by substituting the microcapsules with 0.3% of the same perfume oil used for the encapsulation. This ensured that the quantity of perfume was equal in both products. Two replicates of each product (*microcapsules in LFE* and *reference oil in LFE*) were injected into sealed headspace vials and then allowed to equilibrate for 3 h at room temperature before being analyzed by GC-MS. The GC-MS analyses were performed by sampling the headspace of each vial via SPME with a vial penetration of 25 mm and an extraction time of 1 min at room temperature. Ion extraction of the specific mass for each component was obtained. The SPME fiber was subsequently on-line and thermally desorbed into the GC using a ramp from 40 °C (0.5 min) to 270 °C (0.25 min) at 17 °C/min. The perfume raw materials with a molecular weight between 35 and 300 *m*/*z* were analyzed by GC/MS in full scan mode.

The sum of all the areas under the chromatogram peaks corresponding to the perfume in the microcapsules in LFE and reference oil in LFE were calculated as $Area_{Microcapsules\;in\;LFE\;}$ and $Area_{Reference\;Oil\;in\;LFE}$, respectively.

The *Free Oil %* was then calculated by Equation (2):

(2) $$Free\;Oil\;\%=\frac{Area_{Microcapsules\;in\;LFE\;}}{Area_{Reference\;Oil\;in\;LFE}}\cdot 100$$

The *Free Oil* % is defined as the perfume outside the capsule core. Hence, it indicates the amount of perfume that either was not successfully encapsulated or that had already leaked out in the outer slurry formulation at the time of analysis. The *Free Oil* % values were then calculated in terms of *Encapsulation Efficiency* (EE) by Equation (3):

(3) $$EE\;\%=\frac{100-Free\;Oil\%\;}{100}\cdot 100$$

In synthesis, EE % represents the amount of perfume present in the core compared to the theoretical perfume concentration added during the capsule synthesis.

### 2.7. GC-MS On-Fabric Delivery Assessment

The *On-Fabric delivery* evaluation utilized the P&G standard method TMD01389. Precisely Miele w1714 washing machines for fabric treatment were employed. Each machine was loaded with 3 kg of fabric, consisting of “terry towel cotton fabric tracers” and a mixed variety of fabrics. The terry towel cotton weighed approximately 870 g. The mixed load comprised roughly 1065 g of knitted cotton fabric and 1065 g of polyester–cotton fabrics, maintaining a 50/50 ratio between cotton and polyester. This fabric load also included twenty terry towel tracers.

Before the washing procedure, the machine was thoroughly cleaned. Four ethanol wipes were used: one for the first half of the inox drum, another for the second half, a third for the washing machine’s rubber seal, and the fourth for the detergent drawer. The washing machine door was left open for at least a minute. A cleansing cycle was then run at 95 °C. Before the test treatment, the fabric load underwent two pre-conditioning cycles at 95 °C using the short cotton cycle, each time with 79 g of unscented IEC A Base detergent from WFK Testgewebe GmbH (Brüggen, Germany). Two more 95 °C washes followed this without any detergent.

For the actual test treatment, the fabric was washed on a 30 °C short cotton cycle at a spin speed of 1200 rpm. We used 79 g of the IEC A Base detergent, introduced at the beginning of the cycle in the designated dispenser. Additionally, 40 mL of the LFE fabric treatment composition, as detailed in Table 3, was added to the correct dispenser.

After the washing cycle, the fabric tracers were taken out and line-dried overnight for approximately 12 h in an enclosed space. Two fabric samples, each 4 × 4 cm in size, were cut from two of the terry towel cotton tracers and placed into 25 mL headspace vials. The headspace above these samples was then analyzed using the SPME headspace GC/MS method, as outlined in the *Encapsulation Efficiency* Determination section. The perfume concentration in the headspace is presented in nmol/L.

## 3. Results

The molecular weight between cross-links (cap M cap W sub c) is defined to provide a quantification parameter to characterize the molecular structure of the capsule wall. The MW between Cross-links is a parameter that characterizes the average distance between cross-linking points in the polymer network. Since acrylic monomer conversion is reported to reach full conversion [24,25], it was reasonably assumed that the acrylic conversion was completed. Under this hypothesis, the MW between Cross-links can be calculated by dividing the average molecular weight of the monomers ($M_{av}$) by the number of acrylic groups per molecule (c) [26], as shown in Equation (4):

(4) $$MW_{c}=\frac{M_{av}}{c}$$

In Table 4, the batches of capsules are characterized in terms of their molecular structures, such by $MW_{c}$ and *HSP Distance*. The volume-weighted mean diameter (VW mean diameter) is reported as calculated via AccuSizer 780 AD, which leverages single-particle optical sensing (SPOS) [27].

**Figure 5:**
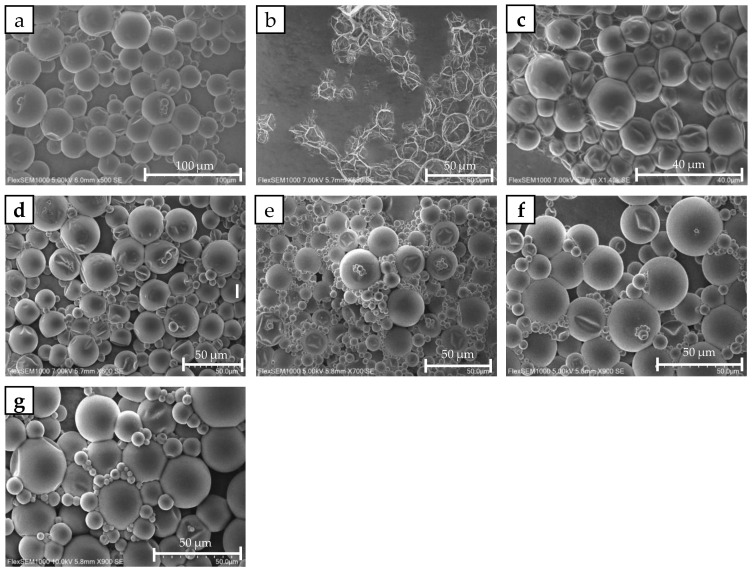
SEM photographs of the microcapsule slurries: (**a**) CAP|1|0|0, (**b**) CAP|0|1|0|, (**c**) CAP|0.3|0.7|0|, (**d**) CAP|0.5|0.5|0|, (**e**) CAP|0|0|1|, (**f**) CAP|0.3|0|0.7|, and (**g**) CAP|0.5|0|0.5|.

The morphology of the prepared batches was characterized by scanning electron microscopy (SEM) using a FlexSEM 1000 from Hitachi (Chiyoda City, Tokyo, Japan). The images for the batches of Table 4 are shown in Figure 5.

When the capsules with the highest $MW_{c}$ (CAP|0|1|0|) were included in the SEM environment, they completely collapsed, presenting a highly distorted morphology. In contrast, the other capsules with lower $MW_{c}$ exhibited a more intact appearance. This visual discrepancy suggests that CAP|0|1|0| is characterized by poor encapsulation quality. The reason for this poor structural integrity will be discussed in the Discussion section.

**Figure 6:**
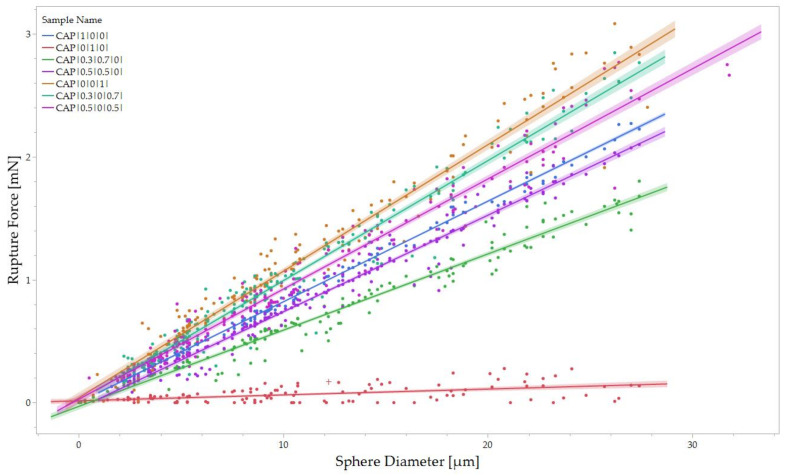
Rupture force for each sample population plotted in function of the diameter. Linear regression and 95% confidence intervals are reported for each sample.

The batch samples were then mechanically characterized with the abovementioned nanoindentation protocol. The rupture force of the individual capsules versus their diameters was then plotted in Figure 6.

A linear relationship between the force required to break a capsule and its diameter can be evidenced, as highlighted in Figure 6. In other words, the size of the capsule has a direct impact on its mechanical properties. Larger capsules have a greater surface area and volume compared to smaller ones. This increased surface area allows for a more extensive stress distribution when external forces are applied. As a result, the load is distributed over a larger area, reducing the stress concentration on any particular point within the capsule.

Consequently, the force required to cause rupture increases in proportion to the diameter of the capsule. Furthermore, the internal volume of the capsule also influences its mechanical properties. This higher internal volume for larger capsules can contribute to the capsule’s overall structural stability and strength, requiring a higher force to break the capsule.

A first-order linear regression analysis with a zero intercept was conducted to predict the value of rupture force as a function of the capsule diameter. As a result, the relationship between rupture force and diameter is highlighted by Equation (5):

(5) Rupture Force=K∗Diameter

The zero intercept was chosen to interpolate the boundary condition for an infinitely small capsule, equivalent to a capsule with a diameter of zero, which would theoretically have an infinitely small rupture force. In Equation (5), the variable *K* represents the linear regression slope, establishing the relationship between rupture force and diameter. As such, this ratio is the *average Rupture Force per unit diameter* (*RFD*), representing the change in rupture force per unit diameter. The term average refers to the interpolation over the distribution of diameters, making *RFD* a material characteristic property of each tested wall composition.

*RFD* is believed to provide a more realistic measure of a microcapsule’s ability to withstand compressive stress before rupturing, compared to rupture stress, as defined by Long et al. [28]. The rupture stress depends on specific geometric elements, such as the surface area before deformation, while *RFD* remains constant within the same capsule population, regardless of the microcapsule size. Moreover, using the rupture stress (given its bulk-equivalent parameter nature) might lead to incomplete interpretations:

- The stress distribution within the membrane might not be uniform [29]. Due to geometry and material properties, different membrane regions might experience different stress levels. Using simple average stress might not accurately capture this non-uniform distribution.
- Polymeric materials often have complex microstructures that can influence their mechanical behavior [30]. A bulk-based calculation might overlook microstructural effects that significantly affect fracture toughness at the micro-scale.
- At the micro-scale, the membrane size becomes comparable to the characteristic length scales of the material microstructure. This can lead to size-dependent mechanical behavior, such as enhanced increased brittleness. Simply scaling down bulk properties might not account for these size effects.

In summary, using the rupture stress alone might oversimplify the polymeric membrane’s mechanical behavior during compression testing at the micro-scale. The *RFD* parameter, instead, is not related to the computation of the stress distribution in the membrane at incipient failure; it can provide several advantages for this specific application:

- Calculating stress distribution within a complex micro-scale membrane can be challenging. It might require advanced techniques like finite element analysis, which can be computationally intensive and require accurate material property data [31,32,33]. Using the rupture load is simpler, making it a practical choice when detailed stress analysis is complex.
- The *RFD* provides a conservative estimate of the material’s strength. It represents the maximum load the population of microcapsules can withstand before failing.
- Comparative analysis: The RFD provides a direct and easy-to-understand metric when comparing different populations. It allows, indeed, quick assessment of which compositional population is stronger or more resistant to rupture.

An example illustrating the significance of *RFD* can be demonstrated through the influence of wall thickness. As the capsule consists of a core/wall structure, the wall thickness plays a crucial role in determining the overall strength of the capsule. *RFD*, being a holistic material characteristic property, considers the contribution of the wall thickness to the capsule’s overall strength. This allows *RFD* to effectively capture the variations in wall thickness within the population of capsules.

In summary, *RFD* comprehensively characterizes the material’s behavior under the incipient loading limit relative to the diameter.

In Table 5, the *RFD* is reported for each sample along with the standard error associated with the calculation of *RFD* via the regression slope between rupture force and diameter.

The capsule batches were analyzed for their performance regarding *Encapsulation Efficiency* and *On-Fabric delivery*.

The results are reported in Table 6.

**Figure 7:**
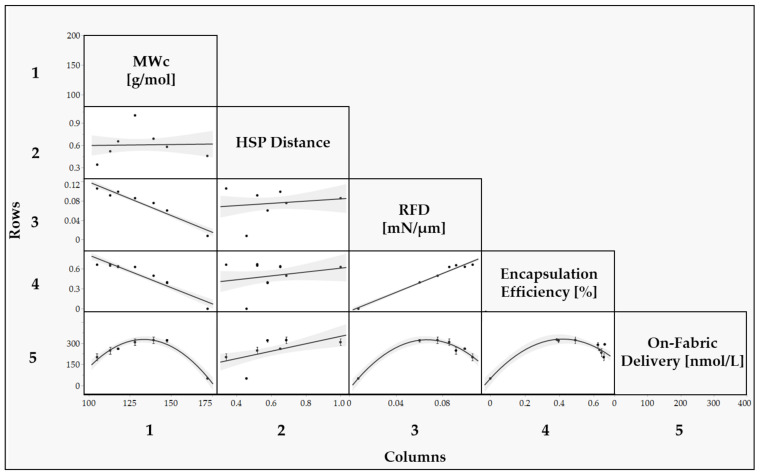
Scatter plot matrix between the chemical structure variables ($MW_{c}$, *HSP distance*) and performance variables (*RFD*, *Encapsulation Efficiency* and *On-Fabric delivery*). The structure–performance correlations are presented as follows: Row 1 and Column 1 represent the correlations comprising the molecular weight between cross-links ($MW_{c}$); Row 2 and Column 2 represents the correlations comprising the *HSP distance*; Row 3 and Column 3 represent the correlations comprising the *RFD*; Row 4 and Column 4 represent the correlations comprising the *Encapsulation Efficiency %*; Row 5 and Column 5 represent the correlations comprising *On-Fabric delivery*.

In Figure 7, a multivariate analysis was conducted by generating a scatterplot matrix to assess the correlation between the parameters related to the chemical structures of the monomer mixture employed ($MW_{c}$, *HSP distance*), and the parameters related to the performance of the capsules (*RFD*, *Encapsulation Efficiency*, *On-Fabric delivery*). A logarithm transformation was applied for the *Encapsulation Efficiency %* values to address the data’s non-normality, as explained in the Supporting Information Document.

## 4. Discussion

The correlation between *MW_c_* and *RFD* reveals that as *MW_c_* increases, the force necessary to rupture each capsule decreases, supported by an R^2^ value of 0.9.

A lower *MW_c_* implies a denser cross-link network within the encapsulation shell. This dense interconnectedness augments capsule resilience against external forces, necessitating greater force to breach the capsules. Conversely, a higher *MW_c_* suggests a looser network, diminishing the capsule’s resistance. The polymer’s activity and cross-linking density significantly impact the elastic modulus and hardness, driven by the microstructural alterations stemming from these parameters. The activity of the polymer, often linked to the glass transition temperature (T_g_), dictates polymer chain mobility. Enhanced polymer activity, either through elevated temperatures or increased chain flexibility, boosts segmental mobility, leading to a decline in elastic modulus and hardness. This is because polymer chains can deform more readily under stress during nanoindentation [34,35].

Conversely, cross-linking density profoundly impacts the mechanical properties of polymers. Cross-links serve as physical barriers, curtailing chain mobility and augmenting polymer network rigidity. A surge in cross-linking density typically raises both the elastic modulus and hardness since the network is less susceptible to deformation, resulting in a more rigid and harder material [36,37,38].

In summary, $MW_{c}$ is a robust predictor for both *RFD* and *Encapsulation Efficiency (EE)*, underlining their collinearity. This relationship can be attributed to higher *EE* values, signifying superior encapsulation quality and enhanced mechanical strength. The close association between *RFD* and *EE* suggests potential interchangeability, evidenced by samples such as CAP|0|1|0|, which presented an *EE* of 0% and the lowest *RFD* in the study. Conversely, CAP|0.5|0.5|0| manifested a high *EE* alongside the maximum *RFD*. Nevertheless, it is crucial to acknowledge that *EE* and *RFD* are not entirely interchangeable. For instance, the collinearity is not consistently evident within the *EE*% range of 94% to 96%. Samples like CAP|0|0|1|, CAP|0.3|0|0.7|, and CAP|0.5|0.5|0| demonstrated similar *EE* values, but a discernible difference in *RFD*, underscoring the unique significance of each parameter and necessitating individual consideration.

The *HSP Distance* provides valuable information on the solubility of the monomer mixture in the perfume mixture, which is expected to be a relevant parameter in the encapsulation process since it affects the transfer of the monomer to the interphase during encapsulation. However, in this specific case, solubility does not seem to be a parameter strongly influencing the performance of these samples since the correlation between *EE* and *HSP Distance* was not statistically significant, as highlighted by a low R^2^. Since all the samples, except CAP|0|1|0|, exhibited good morphological characteristics, according to the SEM pictures, and relatively high *EE*, it appears that for all these samples, the *HSP Distance* between the monomer mixture and the perfume mixture is enough to enable a satisfactory transfer of monomer to the interphase, hence a successful encapsulation.

Overall, the higher correlation between *RFD* and *EE* indicates that $MW_{c}$ is the predominant chemical parameter in determining the encapsulation outcome, rather than the *HSP Distance*. This suggests that controlling $MW_{c}$ is crucial for achieving the desired encapsulation results within the molecular structures included in this study.

To further understand the influence of monomer solubility on the capsule, it would be valuable to test monomer differences with higher differences in *HSP Distance*. Similarly, in future studies, it would be worth evaluating perfume leakage over time, as the HSP distance can strongly influence this parameter since the more the perfume is soluble in the wall membrane, the more effortlessly it would permeate through. Lastly, solubility parameters beyond the *HSP Distance*, such as solubility computed via COSMOtherm [39], should be considered for further research to broaden the understanding of monomer–perfume interactions beyond *HSP Distance*.

The relationship between all variables and *On-Fabric delivery* is not linear; instead, a quadratic model is more fitting, attributed to the intricate dynamics of perfume microcapsule delivery to fabrics during washing. This complexity is due to unpredictable factors such as capsule deposition and retention [40]. The deposition and retention of capsules are influenced by the surface affinity between the capsule wall and the fabric, which involves steps not thoroughly investigated in this study. Hence, the correlation appears more intricate than a linear relationship.

Figure 7 underscores that *RFD* is the primary parameter correlating with *On-Fabric delivery*. It implies that washing cycles, encompassing both low-speed and high-speed rotations [41], profoundly impact the mechanical integrity of the capsules. Hence, the mechanical robustness of the capsules directly influences *On-Fabric delivery*. The data reveals a parabolic relationship between *RFD* and *On-Fabric delivery*, suggesting an equilibrium between capsule resilience and perfume discharge.

An increasing relationship for *RFD* values lower than 6.0 × 10^1^ N/mm is observed, while beyond the 6.0 × 10^1^ N/mm threshold, the relationship changes to decreasing. Stronger capsules are believed to resist rupture better and remain intact under the mechanical stress of the washing machine. Their increased strength enables them to withstand rigorous conditions, avoiding premature rupture and maintaining structural integrity. As a result, more capsules can be deposited onto the fabric surface without breaking, leading to a linear rise in *On-Fabric delivery*. However, beyond the optimal balance point of 6.0 × 10^1^ N/mm, further increases in rupture force may not necessarily result in a proportional increase in the number of deposited capsules. Excessive increases in capsule strength can hinder perfume release: indeed, when the capsules become excessively strong, they may be less prone to rupture even under normal usage conditions, thus limiting the release of the encapsulated perfume.

## 5. Conclusions

The proposed multivariate approach facilitated insights into the influence of the wall’s chemical structure on mechanical properties and delivery efficiency.

For acrylic-based microcapsules, a reduction in the $MW_{c}$ of the monomer mixture from 172 g/mol to 100 g/mol led to enhanced performance. Specifically, there was an improvement in *RFD* (rising from 7.7 ± 5.1 × 10^−1^ to 1.1 × 10^2^ ± 9.7 × 10^−1^ N/mm) and *Encapsulation Efficiency* (increasing from 0% to 95.1 ± 0.3%). It is postulated that this trend primarily stems from an augmented cross-linking density. This density serves as a physical barrier, curtailing chain mobility and amplifying the rigidity of the polymer network.

Within the purview of this investigation, samples CAP|1|0|0|, CAP|0.3|0|0.7|, and CAP|0.5|0.5|0|—comprising mixtures of (oligoglycolate diacrylate) ethylene glycol and aromatic hexafunctional urethane acrylate—registered the pinnacle of *On-Fabric delivery* (roughly 300 nmol/L) in the context of *Liquid Fabric Enhancers (LFE)*. The 100% aromatic hexafunctional urethane acrylate system emerged as the most favorable, attributed to its optimal $MW_{c}$ of 128 g/mol. This resulted in the prime *On-Fabric delivery* (307.5 ± 45.7 nmol/L), bolstered by an *RFD* of 8.5 × 10^1^ ± 6.1 × 10^−1^ N/mm and an *Encapsulation Efficiency* of 94.3 ± 0.2%.

By strategically selecting the molecular structure of the capsule wall, primarily through pinpointing the ideal $MW_{c}$ range, it is feasible to manipulate the cross-linking density. This, in turn, impacts the mechanical attributes of the encapsulating shell. Such adjustments are paramount for tailoring the delivery efficiency to suit specific applications.

Consequently, more efficacious microcapsules pave the way for reducing the quantity of perfume and actives in formulation. This offers a more judicious utilization of chemicals, enhancing sustainability, and also ensures the potential for refining other microcapsule attributes, including environmental ramifications and affinity to core materials.

## Figures and Tables

**Table 1:** Chemical structures and MW of acrylate monomers employed.

Name	Structure	MW [g/mol]	# Acrylate Moiety
A	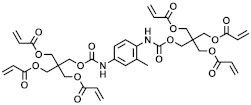	770	6
B	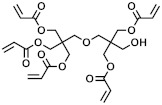	578	5
C	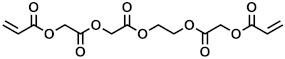	344	2

**Table 2:** Monomer composition of capsules synthesized.

Sample Name	Monomer Mixture Composition			Core:Wall Ratio
	% A	% B	% C	
CAP|1|0|0|	100	0	0	95:5
CAP|0|1|0|	0	100	0	95:5
CAP|0.3|0.7|0|	30	70	0	95:5
CAP|0.5|0.5|0	50	50	0	95:5
CAP |0|0|1|	0	0	100	95:5
CAP |0.3|0|0.7|	30	0	70	95:5
CAP|0.5|0|0.5|	50	0	50	95:5

**Table 3:** LFE formulation comprising microcapsules.

Ingredient	wt.%
Softening active	7.0 × 10^0^
Formic acid	4.5 × 10^−2^
Sodium hydroxyethane diphosphonic acid	7.1 × 10^−3^
Silicone antifoam	2.0 × 10^−3^
Perfume via microcapsules	3.0 × 10^−1^
Water	Balance to 100%

**Table 4:** Characterization of capsule batches in terms of $MW_{c}$ and Hansen solubility distance.

Sample Name	Monomer Mixture			MW between Cross-Links	HSP Distance	VW Mean Diameter [μm]
	% A	% B	% C			
CAP|1|0|0|	100	0	0	128	1	20
CAP|0|1|0|	0	100	0	172	0.46	18
CAP|0.3|0.7|0|	30	70	0	147	0.58	24
CAP|0.5|0.5|0|	50	50	0	139	0.69	24
CAP|0|0|1|	0	0	100	105	0.34	21
CAP|0.3|0|0.7|	30	0	70	112	0.52	22
CAP|0.5|0|0.5|	50	0	50	117	0.65	21

**Table 5:** Mechanical characterization of the different capsule batches.

Sample Name	MW between Cross-Links [g/mol]	RFD [N/mm]
CAP|1|0|0|	128	8.5 × 10^1^ ± 6.1 × 10^−1^
CAP|0|1|0|	172	7.7 × 10^0^ ± 5.1 × 10^−1^
CAP|0.3|0.7|0|	147	6.0 × 10^1^ ± 4.8 × 10^−1^
CAP|0.5|0.5|0|	139	7.5 × 10^1^ ± 5.2 × 10^−1^
CAP|0|0|1|	105	1.1 × 10^2^ ± 9.7 × 10^−1^
CAP|0.3|0|0.7|	112	9.1 × 10^1^ ± 7.4 × 10^−1^
CAP||0.5|0|0.5|	117	9.9 × 10^1^ ± 7.8 × 10^−1^

**Table 6:** Performance characterization of the capsules in terms of *Encapsulation Efficiency* and *On-Fabric delivery*.

Sample Name	Encapsulation Efficiency [%]	On-Fabric Delivery [nmol/L]
CAP|1|0|0|	94.3 ± 0.2	307.5 ± 45.7
CAP|0|1|0|	0	50.02 ± 12
CAP|0.3|0.7|0|	83.8 ± 0.3	319.6 ± 19.5
CAP|0.5|0.5|0|	89.6 ± 0.2	322.1 ± 47.9
CAP|0|0|1|	95.1 ± 0.3	201.3 ± 51.3
CAP|0.3|0|0.7|	94.8 ± 0.2	248.1 ± 49.8
CAP|0.5|0|0.5|	94.3 ± 0.3	260.7 ± 9.6

## Data Availability

The data presented in this study are available on request from the corresponding author. The data are not publicly available due to their deposition on an offline disk, MEMTEC group, URV, Tarragona, Spain.

## Supplementary Materials

The following supporting information can be downloaded at: https://www.mdpi.com/article/10.3390/polym15204158/s1, Paragraph S1: Encapsulation Efficiency, Figure S1: Distribution of values (left-side) and Shapiro-Wilk statistic test on the EE data (right side); Equation (S1). Reference [42] are cited in the Supplementary Materials.

## References

[1] Rowe D.J. (2009). Chemistry and Technology of Flavours and Fragrances.

[2] Lee H., Choi C.H., Abbaspourrad A., Wesner C., Caggioni M., Zhu T., Weitz D.A. (2016). Encapsulation and Enhanced Retention of Fragrance in Polymer Microcapsules. Am. Chem. Soc..

[3] Casanova F., Santos L. (2015). Encapsulation of cosmetic active ingredients for topical application—A review. J. Microencapsul..

[4] Choudhury N., Meghwal M., Das K. (2021). Microencapsulation: An overview on concepts, methods, properties and applications in foods. Food Front..

[5] Dubey R. (2009). Microencapsulation Technology and Applications. Def. Sci. J..

[6] Hakeim O.A., Abdelghaffar F., Haroun A.A. (2020). UV-curable hyperbranched polyester acrylate encapsulation of phthalocyanine pigments for high performance synthetic fabrics printing. Dyes Pigments.

[7] Qiu X., Song G., Chu X., Li X., Tang G. (2013). Preparation, thermal properties and thermal reliabilities of microencapsulated n-octadecane with acrylic-based polymer shells for thermal energy storage. Thermochim. Acta.

[8] Keller M.W., Sottos N.R. (2006). Mechanical properties of microcapsules used in a self-healing polymer. Exp. Mech..

[9] Jyothi N.V.N., Prasanna P.M., Sakarkar S.N., Prabha K.S., Ramaiah P.S., Srawan G.Y. (2010). Microencapsulation techniques, factors influencing encapsulation efficiency. J. Microencapsul..

[10] Vinogradova O.I. (2004). Mechanical properties of polyelectrolyte multilayer microcapsules. J. Phys. Condens. Matter.

[11] Lulevich V.V., Radtchenko I.L., Sukhorukov G.B., Vinogradova O.I. (2003). Mechanical properties of polyelectrolyte microcapsules filled with a neutral polymer. Macromolecules.

[12] Yeo Y., Park K. (2004). Control of encapsulation efficiency and initial burst in polymeric microparticle systems. Arch. Pharmacal Res..

[13] Gray A., Egan S., Bakalis S., Zhang Z. (2016). Determination of microcapsule physicochemical, structural, and mechanical properties. Particuology.

[14] Butt H.J., Cappella B., Kappl M. (2005). Force measurements with the atomic force microscope: Technique, interpretation and applications. Surf. Sci. Rep..

[15] Fernandes P.A., Delcea M., Skirtach A.G., Möhwald H., Fery A. (2010). Quantification of release from microcapsules upon mechanical deformation with AFM. Soft Matter.

[16] Su J.F., Wang X.Y., Dong H. (2012). Micromechanical properties of melamine–formaldehyde microcapsules by nanoindentation: Effect of size and shell thickness. Mater. Lett..

[17] Hayes S.A., Goruppa A.A., Jones F.R. (2004). Dynamic nanoindentation as a tool for the examination of polymeric materials. J. Mater. Res..

[18] Ghaemi A., Philipp A., Bauer A., Last K., Fery A., Gekle S. (2016). Mechanical behaviour of micro-capsules and their rupture under compression. Chem. Eng. Sci..

[19] Mercadé-Prieto R., Allen R., York D., Preece J.A., Goodwin T.E., Zhang Z. (2012). Determination of the Failure Stresses for Fluid-filled Microcapsules that Rupture Near the Elastic Regime. Exp. Mech..

[20] Prieto S.F., Smets J., Jan P., Maria S., Laura O. (2017). Fabric Softener Composition Comprising Encapsulated Benefit Agent. US Patent.

[21] Bakry A.M., Abbas S., Ali B., Majeed H., Abouelwafa M.Y., Mousa A., Liang L. (2016). Microencapsulation of Oils: A Comprehensive Review of Benefits, Techniques, and Applications. Compr. Rev. Food Sci. Food Saf..

[22] Nguon O., Lagugné-Labarthet F., Brandys F.A., Li J., Gillies E.R. (2017). Microencapsulation by in situ Polymerization of Amino Resins. Polym. Rev..

[23] Hansen C.M. (1967). The Three Dimensional Solubility Parameter.

[24] Fiume M.Z. (2002). Final Report on the Safety Assessment of Acrylates Copolymer and 33 Related Cosmetic Ingredients. Int. J. Toxicol..

[25] Capek I., Potisk P. (1995). Microemulsion and emulsion polymerization of butyl acrylate—I. Effect of the initiator type and temperature. Eur. Polym. J..

[26] Wool R.P. (2005). Properties of Triglyceride-Based Thermosets. Bio-Based Polymers and Composites.

[27] Allen T. (1981). Particle Size Measurement.

[28] Long Y., York D., Zhang Z., Preece J.A. (2009). Microcapsules with low content of formaldehyde: Preparation and characterization. J. Mater. Chem..

[29] Zhang J., Oueslati A., Shen W.Q., de Saxcé G. (2018). Exact elastic solution of the axisymmetric and deviatoric loaded hollow sphere. Int. J. Press. Vessel. Pip..

[30] Ulbricht M. (2006). Advanced functional polymer membranes. Polymer.

[31] Sun Y., Peng G., Dou G., Hu Y., Chen P., Zhang T. (2022). A nano-compression model to characterize the elastic properties of core–shell structured microspheres. Thin-Walled Struct..

[32] Sun Y., Peng G., Hu Y., Dou G., Chen P., Zhang T. (2021). Spherical indentation model for evaluating the elastic properties of the shell of microsphere with core-shell structure. Int. J. Solids Struct..

[33] Chen X., Li C., Wei X.X. (2016). Stress analysis of a hollow sphere compressed between two flat platens. Int. J. Mech. Sci..

[34] Beake B.D., Bell G.A., Brostow W., Chonkaew W. (2007). Nanoindentation creep and glass transition temperatures in polymers. Polym. Int..

[35] Odegard G.M., Gates T.S., Herring H.M. (2005). Characterization of viscoelastic properties of polymeric materials through nanoindentation. Exp. Mech..

[36] Deuschle J.K., De Souza E.J., Arzt E., Enders S. (2010). Nanoindentation studies on crosslinking and curing effects of PDMS. Int. J. Mater. Res..

[37] Yiapanis G., Henry D.J., Evans E., Yarovsky I. (2010). Simulations of nanoindentation of polymer surfaces: Effects of surface cross-linking on adhesion and hardness. J. Phys. Chem. C.

[38] Ovsik M., Manas D., Manas M., Stanek M., Reznicek M. (2016). The Effect of Cross-Linking on Nano-Mechanical Properties of Polyamide. Key Eng. Mater..

[39] Hyttinen N., Prisle N.L. (2020). Improving Solubility and Activity Estimates of Multifunctional Atmospheric Organics by Selecting Conformers in COSMO therm. J. Phys. Chem. A.

[40] Manga M.S., Adetomiwa T., Marks S., Gardy J., Blackburn R.S., Russell S.J., York D.W. (2022). Deposition and retention of differently shaped micro-particles on textiles during laundry processing. Powder Technol..

[41] Uz Zaman S., Tao X., Cochrane C., Koncar V. (2021). E-Textile Systems Reliability Assessment—A Miniaturized Accelerometer Used to Investigate Damage during Their Washing. Sensors.

[42] Shapiro S.S., Wilk M.B. (1965). An analysis of variance test for normality (complete samples). Biometrika.

