# Supplementary material for: A Methodology for Multivariate Investigation on the Effect of Acrylate Molecular Structure on the Mechanical Properties and Delivery Efficiency of Microcapsules via In Situ Polymerization
Source: Polymers (Basel). 2023 Oct 19;15(20):4158. doi: 10.3390/polym15204158 (PMC10610868; doi:10.3390/polym15204158)
Supplement: Supplementary file 1 [file polymers-15-04158-s001.zip › polymers-2640867-supplementary.pdf]

Supporting Information

for

A Methodology for Multivariate Investigation on the Effect of Acrylate Molecular Structure on the Mechanical Properties and Delivery Efficiency of Microcapsules via In Situ Polymerization

S1. Encapsulation Efficiency (EE)

The *Encapsulation Efficiency*, as calculated from Equation 3 from the corresponding article, is not normally distributed, as shown on the left side of Figure 1. Moreover, a Shapiro-Wilk statistic test [1] was performed (as shown on the right side of Figure 1), resulting in 0.56. This highlights the non-normality of the *EE* data. This non-normality was primarily attributed to the presence of the 0% value for the sample CAP|0|1|0|.

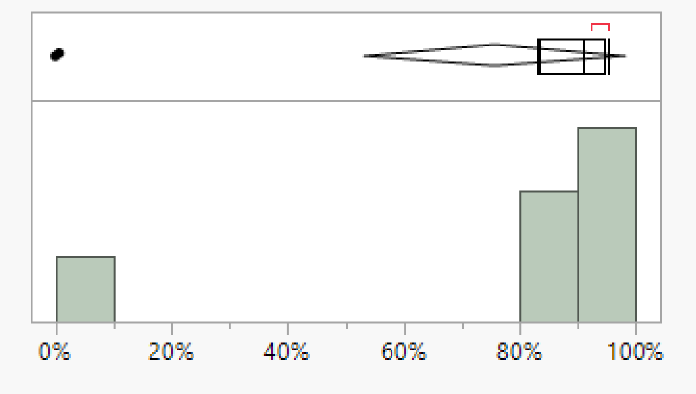

Fitted Normal Distribution

Parameter		Estimate	Std Error	Lower 95%	Upper 95%
Location	μ	0.75835	0.1024957	0.5327585	0.9839415
Dispersion	σ	0.3550556	0.0774795	0.2515196	0.6028411

Measures	
-2*LogLikelihood	8.2029817
AICc	13.536315
BIC	13.172795

Goodness-of-Fit Test

	W	Prob<W
Shapiro-Wilk	0.5615813	<.0001*

	A^2	Simulated p-Value
Anderson-Darling	2.5057209	<.0001*

Note: Ho = The data is from the Normal distribution. Small p-values reject Ho.

Figure S1. *Distribution of values (left side) and Shapiro-Wilk statistic test on the EE data (right side).*

A logarithmic transformation was applied to the *EE%* data to address the non-normality, enabling improved visualization and analysis. Precisely, considering the logarithm quotient rule and to avoid the argument of the log would be zero the $log\ (EE\ \%)$ was calculated as per below equation:

$$log\ (EE\ \%) = \frac{log\ (100\%) - log\ (Free\ Oil\ \%)}{log\ (100\%)} \tag{S1}$$

References

1. M. B. W. S. S. Shapiro, "An analysis of variance test for normality (complete samples," *Biometrika,* vol. Volume 52, no. 3-4, p. 591–611, 1965.
